# Effects of LEGO^®^-Based Neurotherapy on Executive Functions in Children with Autism Spectrum Disorder: A Quasi-Experimental Study

**DOI:** 10.3390/brainsci16060633

**Published:** 2026-06-12

**Authors:** Noemí Cárdenas-Rodríguez, Julieta Griselda Mendoza-Torreblanca, Norma Angélica Labra-Ruiz, Lizbeth Naranjo-Albarrán, Daniel B. LeGoff, Eduardo Espinosa-Garamendi

**Affiliations:** 1 Laboratorio de Neurociencias, Subdirección de Medicina Experimental, Instituto Nacional de Pediatría, Mexico City 04530, Mexico; noemicr2001@yahoo.com.mx (N.C.-R.); julietamt14@hotmail.com (J.G.M.-T.); norma_labra@yahoo.com.mx (N.A.L.-R.); 2 Fundación Cognitive Habilitation, Mexico City 03100, Mexico; 3 Departamento de Matemáticas, Facultad de Ciencias, Universidad Nacional Autónoma de México, Mexico City 04510, Mexico; lizbethna@ciencias.unam.mx; 4 Northeast Neuropsychology, Cheshire, CT 06410, USA; dblegoff@gmail.com; 5 Unidad de Neurohabilitación y Conducta, Servicio de Neurología, Subdireccón de Investigación Médica, Instituto Nacional de Pediatría, Mexico City 04530, Mexico; 6 Departmento de Neuropsicología del Desarrollo y Neurohabilitación, Clínica Cognition, Mexico City 01030, Mexico

**Keywords:** executive functions, LEGO^®^-based neurotherapy, autism spectrum disorder, neurohabilitation, pediatric neuropsychology

## Abstract

**Highlights:**

**What are the main findings?**

- LEGO^®^ Neurotherapy was associated with improvements in executive functions in children with Autism Spectrum Disorder.
- Participants in the LBN group showed improvements in measures related to inhibitory control, working memory, and planning.

**What are the implications of the main findings?**

- LEGO^®^ Neurotherapy may represent a promising complementary intervention to support executive functioning in children with Autism Spectrum Disorder.
- Structured play-based neurotherapeutic approaches may contribute to cognitive rehabilitation in pediatric neurological conditions.

**Abstract:**

**Background:** Children with autism spectrum disorder (ASD) commonly exhibit impairments in executive functioning, which can affect their cognitive and adaptive functioning. Play-based neurohabilitation approaches have been proposed as complementary strategies to stimulate frontal-executive processes. **Objectives:** The primary objective of this study was to assess the effectiveness of LEGO^®^-Based Neurotherapy (LBN) in enhancing executive functioning in children with autism spectrum disorder (ASD). **Methods:** A pilot quasi-experimental pre-post intervention study was conducted in children with ASD. Children voluntarily enrolled either in a LBN program or in a non-intervention comparison group being control (CTRL) group. Executive functions were assessed at baseline and follow-up using the BANFE-3 battery. **Results:** Children participating in the LBN program showed greater improvements in dorsolateral executive-function scores and total executive-function indices compared with CTRL group. These findings suggest a potential association between participation in LBN and executive-function improvement. **Conclusions:** LBN may represent a promising complementary neurohabilitation approach for supporting executive functions in children with ASD.

## 1. Introduction

Autism spectrum disorder (ASD) is a heterogeneous neurodevelopmental disorder characterized by enduring deficits in social communication and social interaction, together with restricted, repetitive patterns of behavior, interests, or activities [1,2]. Major international classification systems define the disorder on the basis of observable behavioral criteria organized into clinical checklists [1,2]. Although ASD diagnosis is currently based primarily on standardized behavioral and clinical assessments, ongoing research continues to explore objective neurobiological and neurocognitive markers that may complement current diagnostic approaches [3,4].

Despite these diagnostic constraints, epidemiological evidence indicates that ASD is among the most prevalent neurodevelopmental disorders worldwide, affecting approximately 1% of children globally [5]. Recent estimates in certain populations report a prevalence as high as 1 in 36 children [6,7]. In Mexico, epidemiological studies have estimated a prevalence of approximately 1 in 115 children, although population-based data remain limited [8,9].

From a neurobiological perspective, ASD is currently conceptualized as a disorder involving the development of distributed brain networks [10,11]. Neuroimaging studies have revealed alterations in regions associated with social cognition and executive control, including the prefrontal cortex, cerebellum, amygdala, and basal ganglia [10,11]. Furthermore, research on brain connectivity has revealed atypical patterns characterized by local hyperconnectivity and reduced long-range connectivity between large-scale brain networks. These alterations may affect the integration of social and cognitive information during development [12,13,14].

Among the neurocognitive domains most consistently affected in individuals with ASD are executive functions, a set of higher-order cognitive processes that encompass working memory, cognitive flexibility, planning, and inhibitory control [15,16]. A substantial body of evidence has demonstrated that individuals with ASD exhibit impairments in tasks requiring adaptation to changing environmental demands, behavioral self-regulation, and the generation and implementation of flexible problem-solving strategies [17]. These alterations have been associated with dysfunction in frontostriatal and frontocerebellar circuits involved in executive control and behavioral regulation [17,18,19,20].

In light of these neurocognitive deficits, considerable efforts have been directed toward the development of therapeutic interventions aimed at strengthening social functioning and executive control processes in individuals with ASD [19,20]. Among play-based structured interventions, LEGO^®^-Based Therapy (LBT) has shown promising results in promoting social interaction, cooperation, and problem-solving skills through guided collaborative activities [21,22]. Complementarily, rehabilitation and cognitive neurohabilitation programs aimed at training executive functions and social cognition have gained increasing attention in applied neuroscience research [23,24,25,26,27].

While traditional LBT primarily focuses on social interaction, communication, and collaborative play in children with ASD, the LEGO^®^-Based Neurotherapy (LBN) approach used in the present study additionally incorporated executive-function challenges within LEGO^®^-based activities [21,22]. These included planning, sequencing, sustained attention, working memory, inhibitory control, problem solving, and cognitive flexibility within a neurohabilitation-oriented framework. Thus, the distinction between LBT and LBN lies in the intentional incorporation of directed executive-function stimulation and experience-dependent cognitive training principles.

Recent studies in neurohabilitation have proposed intervention models focused on strengthening neurocognitive networks involved in executive control and behavioral regulation [28]. These approaches are based on the premise that targeted cognitive training may facilitate the functional reorganization of frontal circuits and improve behavioral adaptation in populations with neurodevelopmental disorders [29]. Within this framework, structured interventions based on construction activities and problem-solving tasks have been developed to stimulate processes such as planning, action sequencing, inhibitory control, and social cooperation through guided activities [30]. Furthermore, studies conducted in various pediatric neurological conditions, including congenital heart disease, epilepsy, and Down syndrome, have shown that neurohabilitation programs targeting executive functions may contribute to improvements in cognitive organization, behavioral self-regulation, and functional adaptation in these populations [31,32,33,34,35].

LBN is grounded in neurohabilitation principles aimed at stimulating executive control, attention, memory, and behavioral regulation through collaborative and guided construction-based activities [31,32]. In the present study, the term “Neurotherapy” is used within a neurohabilitation framework to describe a structured neurocognitive intervention focused on executive and frontal-executive processes through guided cognitive, behavioral, and play-based activities. The term refers to experience-dependent cognitive activation and training rather than direct neuromodulatory intervention [32,33].

Recent studies have applied this intervention model in several pediatric conditions that by its physiopathology presented neurological impairment, including congenital heart disease, epilepsy, obesity and Down syndrome, and have reported improvements in executive functions, attention, memory, and cognitive organization following the use of LEGO^®^ materials and activities [31,32,33,34,35,36]. These findings suggest that neurohabilitation interventions based on structured construction activities may contribute to strengthening frontal networks involved in cognitive control and behavioral regulation in pediatric populations with neurological conditions [31,32,33,34,35]. Accordingly, the present study aimed to evaluate the effects of LBN on executive functioning in children with ASD.

## 2. Methods

### 2.1. Study Design and Ethical Considerations

A quasi-experimental pre–post intervention design was employed involving pediatric patients with autism spectrum disorder (ASD) receiving care at the Neurohabilitation and Behavior Unit of the Instituto Nacional de Pediatría (INP), Mexico City, Mexico. Children were selected using a non-probability convenience sampling method, and inclusion was contingent upon voluntary participation.

Children were recruited between January 2022 and December 2025. Before inclusion in the study, parents or legal guardians and eligible children were provided with comprehensive information concerning the study objectives, procedures, anticipated benefits, potential risks, and any foreseeable inconveniences related to participation. Written informed consent was obtained from all parents or legal guardians, while assent was obtained from participating children when appropriate according to their age and developmental level. The study protocol received approval from the Institutional Research and Ethics Committee of the Instituto Nacional de Pediatría (INP) on the 18 July 2022 (protocol number 2022/045). All procedures were performed in accordance with institutional ethical guidelines and the principles of the Declaration of Helsinki for research involving human participants. All children had a clinical diagnosis of ASD according to the International Classification of Diseases, 11th Revision (ICD-11). The standardized instruments such as ADOS-2, ADI-R, CARS-2, and SRS-2 were not systematically available for all children because diagnoses had already been clinically established prior to study enrollment. Baseline neuropsychological assessment was performed using the BANFE-3 battery, which evaluates executive function across the orbitomedial cortex (OMC), the anterior prefrontal cortex (APC), and the dorsolateral cortex (DLC) regions, as well as a global executive function index.

### 2.2. Participants

Participants were recruited from children diagnosed with ASD according to the ICD-11, who received care at the Mental Health Service of the Instituto Nacional de Pediatría (INP) and had available electronic medical records. Potentially eligible children were identified through medical record review and contacted by telephone.

Prior to enrollment, parents or legal guardians received detailed information regarding the LBN intervention, study procedures, and assessment protocol. Children whose families elected to participate in the LBN intervention were assigned to the LBN group, whereas children whose families declined participation in the intervention underwent neuropsychological assessments at baseline and six months later and constituted the control (CTRL) group. Consequently, group allocation was not randomized, although outcome assessments were conducted under blinded conditions. Because participation in the intervention was voluntary, the study design remains susceptible to selection bias and limits definitive causal inference despite the implementation of blinded neuropsychological assessment procedures.

**Figure 1:**
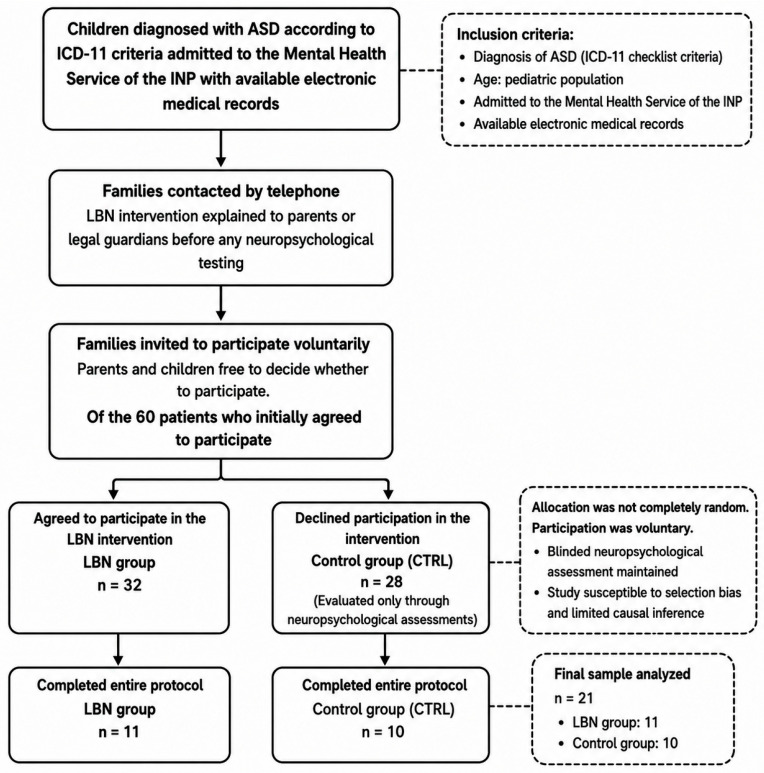
Participant flow diagram showing recruitment, voluntary group allocation, follow-up, attrition, and final sample included in the quasi-experimental analysis.

A total of 60 children were initially enrolled in the study. Of these, 21 completed the full study protocol, including 11 children in the LBN group and 10 children in the CTRL group. The high attrition observed during follow-up was primarily associated with difficulties maintaining weekly attendance, transportation limitations, scheduling conflicts, and discontinuation of follow-up appointments, factors commonly encountered in longitudinal pediatric neurohabilitation research (Figure 1).

All children were evaluated according to predefined inclusion, exclusion, and withdrawal criteria. Inclusion criteria were: (1) age between 6 and 16 years; (2) a clinical diagnosis of ASD according to ICD-11 criteria; (3) evidence of executive functioning difficulties identified during neuropsychological evaluation; and (4) provision of written informed consent by a parent or legal guardian and assent from the child when developmentally appropriate.

Exclusion criteria comprised severe neurological disorders unrelated to ASD, language or motor impairments that could interfere with participation in the intervention (e.g., severe dyspraxia or dysphasia preventing comprehension of instructions), as well as severe sensory or motor limitations that precluded engagement in structured cognitive activities.

Children were withdrawn from the study under the following conditions: (1) voluntary withdrawal at any time after providing informed consent; (2) absence from intervention sessions or follow-up assessments for a period exceeding four weeks after the scheduled date; or (3) failure to complete the neurotherapy intervention protocol.

The baseline characteristics of the pediatric patients with ASD are presented in Table 1. A total of 21 children were included, with 10 in the CTRL group and 11 in the LBN group. All the children were male. The mean age was 10.27 ± 3.01 years in the CTRL group and 8.7 ± 2.45 years in the intervention group. Most children were in primary school. Some received additional therapies, and leisure activities varied across groups. A minority were under pharmacological treatment. Children may be included in more than one category (Table 1).

### 2.3. Instruments

#### 2.3.1. Neuropsychological Battery of Executive Functions (BANFE-3)

Executive functioning was assessed using the Neuropsychological Battery of Executive Functions, Third Edition (BANFE-3), a standardized instrument developed and validated for the Mexican population. The BANFE-3 evaluates executive functions associated with prefrontal cortex functioning and provides a total executive functioning score as well as domain-specific indices related OMC, APC, and DLC functions.

The OMC index assesses processes related to inhibitory control, behavioral regulation, and decision-making. The APC index evaluates higher-order cognitive processes, including abstract reasoning, metacognition, and metamemory. The DLC index assesses working memory, planning, cognitive flexibility, verbal fluency, and visuospatial executive processes.

Raw scores are converted into age-adjusted standardized scores (M = 100, SD = 15). According to the BANFE-3 normative criteria, performance is classified as high normal (≥116), normal (85–115), mild impairment (70–84), or severe impairment (≤69). Previous studies have reported adequate psychometric properties for the BANFE-3, including internal consistency coefficients (Cronbach’s alpha) exceeding 0.80 [36].

#### 2.3.2. Evaluation of LBN Performance

Participant performance during the LBN intervention was assessed using a structured Likert-type rating scale developed to evaluate neuropsychological functions targeted by the intervention and related to the executive function domains assessed by the BANFE-3 [37]. Performance on each activity was rated using a four-point scale: 0 = unable to complete the task; 1 = completed the task with substantial assistance or difficulty; 2 = completed the task independently; and 3 = completed the task accurately and efficiently. Previous studies using this instrument have reported satisfactory reliability indices, with coefficients ranging from 0.80 to 0.90 and adequate interrater agreement [31,32,33,34,35]. For each participant and intervention session, a performance percentage score was calculated by summing the scores obtained across all activities, multiplying the result by 100, and dividing by the maximum possible score. LBN performance scale has been previously applied and reported in pediatric neurohabilitation studies involving children with congenital heart disease and epilepsy [31,32,33,34].

### 2.4. General Procedure

Following enrollment, demographic and clinical information was collected from all children. Baseline executive functioning was assessed using the BANFE-3, which required approximately 60 min to administer.

In the CTRL group, a follow-up neuropsychological assessment was conducted six months after baseline. In contrast, children assigned to the LBN group completed between 12 and 15 intervention sessions according to individual therapeutic progress. Sessions were conducted once weekly, lasted approximately 60 min, and were followed by a post-intervention neuropsychological assessment at six months. After completing the intervention phase, children received clinical follow-up on a biweekly basis and subsequently on a monthly basis until the post-intervention assessment.

Children who initiated the LBN program did not begin additional neuropsychological or behavioral interventions during the active intervention phase. Concurrent therapies established prior to study enrollment remained clinically stable throughout the study period, as verified through institutional follow-up records and parental reports. All intervention and assessment procedures were conducted in a dedicated clinical setting with adequate lighting, a work table, and age-appropriate seating for children. Seating was also provided for the therapist delivering the intervention and the evaluator responsible for recording performance during the activities. Interventionists and evaluators were trained in neuropsychology, cognitive behavioral therapy, and LBN. Evaluators responsible for outcome assessments remained blinded to group allocation throughout the study period to preserve assessment blinding.

### 2.5. Sessions of Intervention Through LBN

The LBN intervention consisted of a structured sequence of sessions designed to progressively stimulate executive and cognitive functions through construction and programming activities. Initially, the sessions focused on familiarizing children with the LEGO^®^ materials and establishing therapeutic interactions through free play, simple assembly tasks, and basic robotic programming. Subsequently, activities were oriented toward the stimulation of working memory, visuospatial processing, selective attention, and inhibitory control through structured construction tasks and guided problem-solving challenges. As the intervention progressed, tasks increased in complexity and incorporated planning, risk selection, sequential organization, and cognitive flexibility through robot assembly, programming activities, and classification exercises. Finally, advanced sessions emphasized the integration of executive functions and cognitive monitoring by engaging children in complex problem-solving tasks that required the coordination of memory, attention, and planning processes. All activities were implemented using LEGO^®^ Education sets and progressively adapted according to the children’s performance and cognitive progress [31,32,33,34,35].

The LBN included sessions with programming, working and visuospatial memory, inhibitory control and integration of follow-up and executive functions integration. These sessions gradually stimulate areas of the cerebral cortex, cognitive habilitation and developmental monitoring. A more detailed description of the therapeutic structure and neurohabilitation principles underlying the LBN intervention has been previously reported in earlier studies conducted by our group (Table 2) [31,32,33,34,35].

### 2.6. Statistical Analysis

The dependent variables were the executive function indices obtained from the BANFE-3, including the OMC, APC, DLC and total executive functioning scores. Between-group differences in post-intervention outcomes were evaluated using analysis of covariance (ANCOVA) [38,39] with baseline scores included as covariates. Bonferroni-adjusted post hoc comparisons were performed when appropriate. Changes in performance were calculated as gain scores (posttest minus pretest values) and analyzed using the Wilcoxon rank-sum test [40]. Moreover, effect size measures were computed [41]. Statistical analyses were conducted using R software (version 3.5.2). Graphical representations were generated using R library ggplot2 [42] and effect size measured were computed with the R library effectsize [43]. Statistical significance was established at *p* ≤ 0.05.

## 3. Results

### 3.1. Cognitive Evaluations of Participants

The standardized executive function scores obtained with the BANFE-3 are presented in Table 3 and Table 4 for the CTRL and LBN groups, respectively. In the CTRL group, OMC scores decreased from 79.0 ± 29.4 at baseline to 72.2 ± 27.3 at follow-up. The mean of APC scores remained relatively stable, changing from 79.1 ± 21.0 to 80.0 ± 27.0, whereas that mean of DLC scores increased slightly from 67.7 ± 16.2 to 68.3 ± 17.5. The total mean of executive function score showed minimal variation, changing from 66.8 ± 16.8 to 66.5 ± 17.0.

In the LBN group, the mean of OMC scores increased from 76.5 ± 33.0 at baseline to 86.6 ± 29.2 at follow-up. The mean of APC scores decreased from 89.7 ± 32.9 to 85.0 ± 25.1, whereas DLC scores increased from 65.7 ± 18.5 to 83.3 ± 27.2. The total mean of executive function score increased from 66.4 ± 20.7 to 81.9 ± 30.6.

Descriptive comparisons suggested greater variation in executive function scores over time in the LBN group than in the CTRL group. The statistical significance of these differences was subsequently evaluated using inferential analyses.

### 3.2. Performance of LBN in Children with ASD

**Figure 2:**
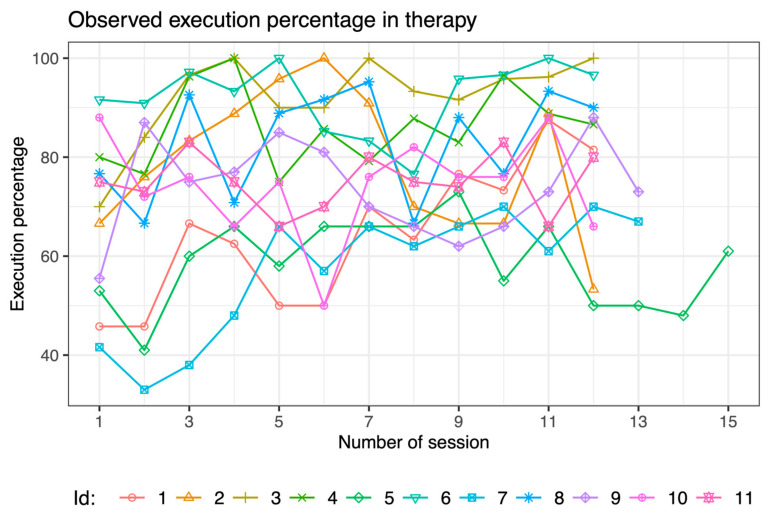
Evaluation of LBN performance by session in children with ASD. Each line is colored according to the participant identifier (Id).

The mean percentages of LBN performance by session for children with ASD are shown in Figure 2. Overall, the data exhibit moderate variability across sessions, reflecting individual differences in engagement and performance.

Despite this variability, an overall positive trend can be observed, with several children reaching high performance levels (close to or at 100%) in later sessions. These findings suggest that continued exposure to LBN may support improvements in task engagement, social interaction, and structured play skills among children with ASD.

### 3.3. Effects of LBN on Children with ASD

**Figure 3:**
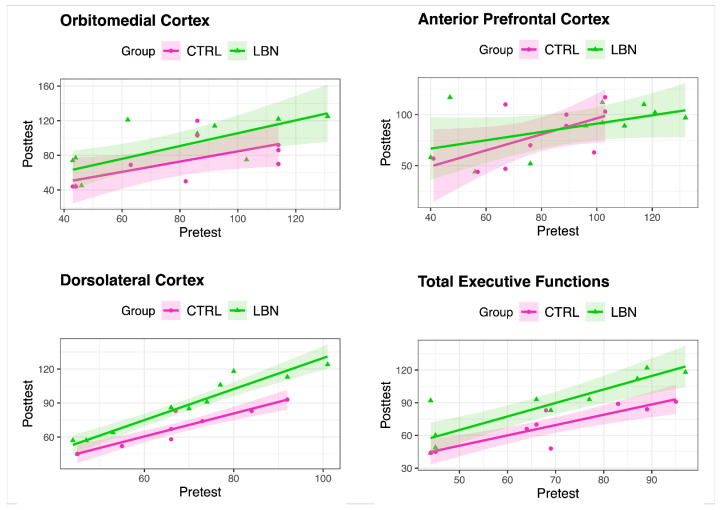
Relationship between pretest and posttest executive function scores assessed with the BANFE-3 in the CTRL and LBN groups. CTRL = control group; LBN = LEGO^®^-Based Neurotherapy group. CTRL, n = 10; LBN, n = 11.

Figure 3 presents the relationships between baseline and post-intervention BANFE-3 scores for the OMC, APC, DLC, and total executive function indices in the CTRL and LBN groups. The graphs present the data points colored by group, the fitted regression lines (solid lines) for both scores by group, and their 95% confidence intervals (shaded areas). In general, the scatterplots show a linear relationship between the pretest and posttest scores, with increasing trends in both groups. The estimated line for the LBN group is over the estimated line for the CTRL group, except for APC, where the estimated lines are crossed and display statistically significant differences in the DLC area and total executive functions; i.e., their estimated 95% confidence intervals are displayed without overlapping.

**Figure 4:**
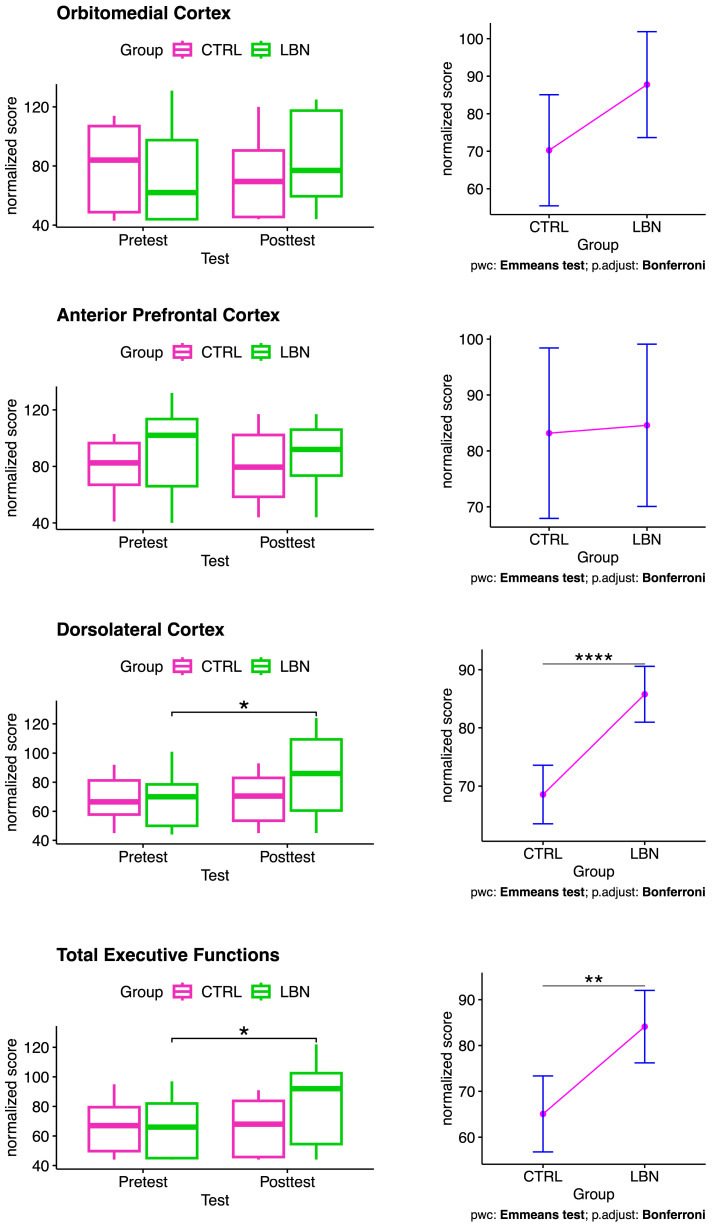
Effect of LBN in children with ASD as evaluated by the BANFE-3 test. Box plots on the left show changes in scores in the CTRL and intervention groups. Higher post-intervention scores were observed in the LBN group in the DLC and total executive functions. ANCOVA test; * *p* ≤ 0.050. The graphs on the right show the pairwise comparisons (pwc) and the estimated marginal means (Emmeans) of the posttest scores by group under the mean of the pretest. ANCOVA followed by the Bonferroni post hoc correction with correction for multiple testing; **** *p* ≤ 0.0001, ** *p* ≤ 0.005. CTRL = control; LBN = LEGO^®^-based Neurotherapy. CTRL n = 10; LBN n = 11.3.4. Gain Score Analysis.

BANFE-3 scores obtained at baseline and post-intervention were compared between the CTRL and LBN groups. Statistical analyses revealed an increase in the LBN group in all evaluated functions, with the exception of APC; i.e., the estimated marginal means (emmeans) increase for the LBN group in comparison to the CTRL group, except for APC, where they are similar (Table S1 in Supplementary Materials); in specific, for the differences Posttest-Pretest, for OMC emmeans = 1.79 (*p*-value = 0.089), for APC emmeans = 0.14 (*p*-value = 0.891), for DLC emmeans = 5.19 (*p*-value < 0.001), and for total executive functions emmeans = 3.49 (*p*-value = 0.003). In addition, the ANCOVA results showed that participants in the LBN group exhibited higher post-intervention scores than those in the CTRL group, with statistically significant differences in the DLC area and total executive functions; i.e., their estimated 95% confidence intervals are displayed without overlapping (Figure 4), and having large effect sizes (Table S1 in Supplementary Materials); in specific, the F test for ANCOVA, and the eta squared (η^2^) and epsilon squared (ε^2^) effect sizes resulted in the following values, for OMC F = 3.22 (*p*-value = 0.089) and small effect size (η^2^ = 0.05, ε^2^ = 0.09), for APC F = 0.02 (*p*-value = 0.891) and small effect size (η^2^ = 0.0, ε^2^ = 0.04), for DLC F = 26.97 (*p*-value < 0.001) and large effect size (η^2^ = 0.50, ε^2^ = 0.53), and for total executive functions F = 12.18 (*p*-value = 0.003) and large effect size (η^2^ = 0.34, ε^2^ = 0.40). Moreover, model diagnostic metrics and statistical tests show that ANCOVA assumptions are met (Table S2 in Supplementary Materials), i.e., linear relationship between the pretest and posttest scores, homogeneity of regression slopes, normality of residuals, homoscedasticity, and there are not outliers neither influence observations.

### 3.4. Analysis of the Gain Score

**Figure 5:**
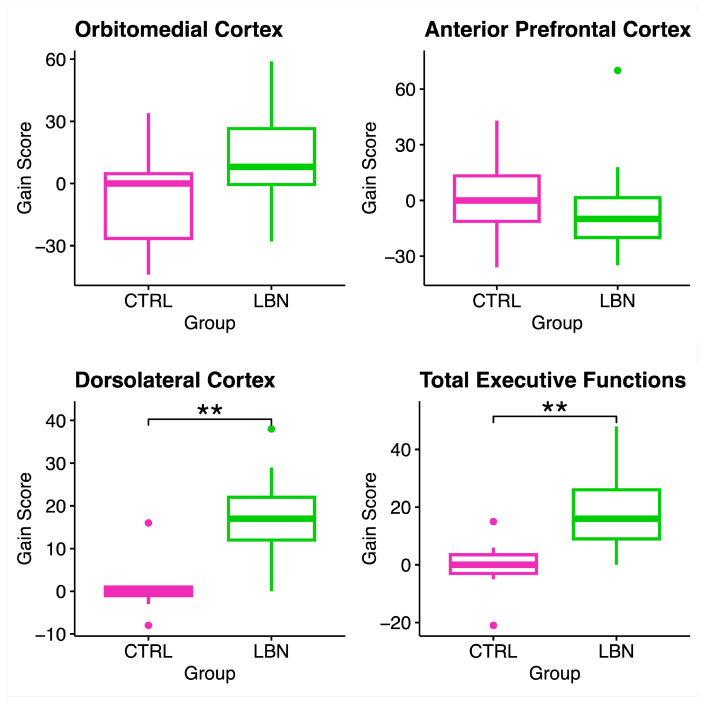
Gain scores (posttest − pretest) for BANFE-3 executive function indices in the CTRL and LBN groups. Significant between-group differences were observed for the DLC and total executive function indices. Wilcoxon rank-sum test; ** *p* ≤ 0.01. CTRL = control group (n = 10); LBN = LEGO^®^-Based Neurotherapy group (n = 11).

Another approach to evaluate the effects of LBN in children with ASD is to compare gain scores, defined as the difference between posttest and pretest performance (gain score = posttest − pretest). The statistical analysis revealed significant differences between the CTRL and LBN groups, having large effect sizes, in DLC and total executive functions (Figure 5, and Table S3 in Supplementary Materials); in specific the Wilcoxon rank sum test and the “r” effect size resulted in the following values, for OMC W = 35.5 (*p*-value = 0.179) and moderate effect size (r = 0.30), for APC W = 68 (*p*-value = 0.378) and small effect size (r = 0.20), for DLC W = 9 (*p*-value = 0.001) and large effect size (r = 0.71), and for total executive function W = 15 (*p*-value = 0.005) and large effect size (r = 0.62).

With respect to the DLC domain, the CTRL group had a median gain score close to zero, with limited variability, whereas the LBN group had notably higher median values and greater dispersion, indicating a more substantial improvement following the intervention. Similarly, for total executive functions, the CTRL group demonstrated minimal changes between the pretest and posttest, whereas the intervention group showed a marked increase in gain scores, with higher median values and wider interquartile ranges.

In contrast, no statistically significant differences were observed between groups in the APC or OMC domains. Overall, the boxplot distributions indicate that compared with the CTRL group, the LBN group achieved greater improvements, particularly in DLC executive functioning and global executive performance.

## 4. Discussion

Our findings are consistent with the hypothesis that structured play-based neurohabilitation may be associated with improvements in executive-function outcomes in children with ASD. Executive dysfunction is among the most consistently reported types of cognitive alteration in individuals with ASD and affects core executive processes, including working memory, inhibitory control, planning, and cognitive flexibility [15,17]. These impairments have been linked to atypical functioning of frontostriatal and frontocerebellar circuits involved in cognitive control and behavioral regulation [16,17,18]. Within this framework, the improvements observed in the LBN group, particularly in DLC scores, could reflect enhanced efficiency in neural systems supporting working memory, planning, and flexible problem solving. To date, studies on the use of LBN are lacking; however, some studies have shown that LBN improves some skills in children with ASD. One study reported improvements in social behavior among 18 children with ASD, with gains maintained over time [44]. Another study revealed that LEGO^®^-based interventions did not significantly improve social skills in 6 kindergarten children with ASD, but individual improvements were noted [45]. Previous studies have also reported modest improvements in social and emotional functioning among children with ASD [46]. In another study, reductions in maladaptive behaviors have also been reported among children with high-functioning autism and Asperger syndrome [27].

The intervention employed in this study is grounded in neurohabilitation principles that emphasize repeated activation and progressive training of cognitive processes to promote functional reorganization of neural networks [24,25]. LBN incorporates structured construction tasks, sequencing, and problem-solving activities, which require the coordinated engagement of executive functions. These findings are consistent with previous studies indicating that targeted cognitive training may support frontal network functioning and adaptive behavior in children with neurodevelopmental conditions [24,28].

These findings are also consistent with previous studies of LEGO^®^-based interventions that have reported benefits in social interaction, cooperation, and problem-solving skills among children with ASD [21,22,23,24]. A study using LBT revealed that in autistic spectrum children, this intervention improved motivation and contact and overcoming autistic symptoms of aloofness and rigidity [21]. The authors suggested that the LBT has potential benefits for related cognitive skills such as executive functions [21,22]. Another study reported improvements in creativity, fine motor skills, motivation, concentration, and social functioning, together with reductions in perceived stress among children with ASD participating in LBT [47]. Although traditional LEGO^®^-based interventions have primarily focused on social skills, the present findings provide preliminary evidence supporting their potential application within neurohabilitation programs targeting executive functions. One study suggested that LBT leverages intense interest in teaching skills related to executive functions [48]. Similar improvements in cognitive domains have been reported in other pediatric populations with LBN, including children with epilepsy, obesity, congenital heart disease, and Down syndrome [31,32,33,34,35].

In general in the neurohabilitation, the CTRL group showed minimal and heterogeneous changes in executive function scores, which are consistent with developmental variability and cortical maturation during childhood. The more consistent changes observed in the intervention group suggest a possible association between participation in LBN and greater improvements in executive-function measures than those observed in the CTRL group. Changes in the APC domain were less consistent than those observed for the DLC and total executive function indices. This finding could be related to the greater complexity of metacognitive and abstract reasoning processes associated with anterior prefrontal functioning and the possibility that these functions require longer intervention periods or more targeted cognitive strategies to achieve measurable change. Overall, the findings support the potential value of structured, experience-dependent neurohabilitation approaches aimed at stimulating executive function processes through sustained cognitive engagement and guided problem-solving activities. In relation to the variability across session observed in LBN performance several factors may explain this dispersion. We suggest that first, variability in social communication and cognitive flexibility among children with ASD may have influenced their ability to follow instructions and complete tasks consistently across sessions. Some children required additional time or support to adapt to the structured activities, which may have affected performance levels in certain sessions. Second, fluctuations in attention, sensory sensitivity, and behavioral regulation likely contributed to changes in performance. In some sessions, children may have experienced overstimulation or reduced motivation, leading to lower performance percentages. Third, inconsistencies in attendance or interruptions in therapy continuity may have affected progress. As observed, missed sessions or breaks in routine can result in temporary declines in performance, followed by recovery in subsequent sessions.

The principal limitation of this study is the small sample size, which may limit the generalizability of the findings and reduce statistical power. Although large samples are generally needed to ensure statistical significance, the use of ANCOVA can improve the precision of estimations, when assumption behind ANOVA are met, and correlations between baseline and outcome are adequate. Related to ANCOVA, previous studies suggest that sample sizes between 8 and 20 children may be sufficient to detect significant differences [49] and more recent studies indicate that under certain conditions, even small samples (~10 subjects) may be adequate, depending on the effect size R^2^ [50]. These results should be interpreted with caution and require validation in studies with larger samples.

Additional limitations include the non-randomized group allocation and the possibility of selection bias associated with voluntary participation. Consequently, the findings should be interpreted with caution and confirmed in larger, preferably randomized, studies.

## 5. Conclusions

In this quasi-experimental study, participation in LBN was associated with positive changes in executive-function measures, particularly in domains related to dorsolateral prefrontal functioning and overall executive performance. These findings suggest the potential utility of structured play-based neurohabilitation strategies for engaging frontal-executive processes and supporting cognitive development in children with ASD. However, given the non-randomized design, small sample size, high attrition, and passive control condition, the findings should be considered preliminary and require confirmation in larger controlled studies. Future research should further investigate the long-term effects, generalizability, and scalability of this intervention in more diverse clinical samples.

The results suggest the potential utility of structured play-based neurotherapeutic approaches as complementary interventions for enhancing executive functioning in children with ASD. These findings are consistent with the emerging literature on neurohabilitation approaches aimed at promoting cognitive development and functional adaptation in individuals with neurodevelopmental disorders.

## 6. Limitations

Several limitations should be considered when interpreting these findings. First, the study used a quasi-experimental design without random allocation, which increases susceptibility to selection bias and limits causal inference. Second, the sample size was small and attrition during follow-up was high, reducing statistical power and limiting generalizability. A substantial proportion of participant dropout was associated with socioeconomic and logistical barriers frequently encountered in public pediatric neurohabilitation settings, including limited financial resources, transportation difficulties, long travel distances to attend weekly sessions, and challenges maintaining regular follow-up appointments. Many children had also been referred from multiple healthcare or educational centers due to the absence of specialized local services, which further complicated continuity of care. In several cases, families assigned to the CTRL group were unable to continue attending follow-up visits because of economic limitations, although they remained interested in obtaining neuropsychological assessments to support school placement or referral to nearby treatment centers. Additionally, the CTRL group was passive and did not include an active attention-matched comparison condition. Therefore, the findings should be interpreted as preliminary and hypothesis-generating.

## Figures and Tables

**Table 1:** Characteristics of pediatric patients with ASD.

Variable	Characteristics	CTRL Group	LBN Group
Sex ^1^	Male	10 (100%)	11 (100%)
Age ^2^	Years	10.27 ± 3.01	8.7 ± 2.45
School Grade ^1^	Primary	8 (80%)	8 (72.7%)
	Secondary	2 (20%)	3 (27.3%)
Other Therapies ^1^	Psychological	3 (30%)	2 (18.2%)
	Language	2 (20%)	1 (9.1%)
	Occupational	1 (10%)	0 (0%)
	Physiotherapy	3 (30%)	0 (0%)
Leisure Activities ^1^	Tablet	3 (30%)	1 (9.1%)
	Free game	3 (30%)	4 (36.4%)
	Sports	1 (10%)	4 (36.4%)
Pharmacological Treatment ^1^	Fluoxetine	1 (10%)	0 (0%)
	Methylphenidate	1 (10%)	3 (27.3%)
	Risperidone	0 (0%)	1 (9.1%)

^1^ Counts and percentages. ^2^ Mean ± standard deviation. Children may be included in more than one category.

**Table 2:** Intervention of cognitive functions with LEGO^®^-based Neurotherapy.

Sessions	Objectives	Tasks	Sets
(1) Free Play and Introductory Programming	Initial interaction, familiarizing the patient with the material and establishing therapeutic rapport.	Identify colored blocks and free assembly Initial robot assembly and programming In severe cases, start assembling animal sets Simple machine assembly Programming robotic challenges involving forward and backward movement sequences at 10 s intervals.	LEGO^®^ DUPLO^®^ bricks WeDo 2.0^®^ Set LEGO^®^ Simple Machines Set/Coding Express Bingo LEGO^®^ Education Bingo Set Pre-assembled memory blocks Colored blocks
(2) and (3) Working Memory and Visuospatial Memory	Gradually stimulates areas of the cerebral cortex to improve selective attention, inhibitory control, and short-term memory	Begin with working memory training and assembling blocks of two or three pieces Visuospatial memory training activities. Working-memory templates using blocks of the same color. Turn-taking activities and challenge configuration. Assembling and programming of robots with challenges Simple disassembly and reassembly of a machine or robot challenge without the assistance of a template or therapist.	LEGO^®^ DUPLO^®^ bricks WeDo 2.0^®^ Set/Spike essential LEGO^®^ Simple Machines Set Pre-assembled block templates +Cafe Colored blocks of different size
(4) to (9) Working memory, inhibitory control, decision-making and planning	Stimulation of cerebral cortex areas to improve the effort investment process of related functions	Begin with working memory exercises and assembling blocks of three to four pieces Stimulate with visuospatial working memory template and color the blocks Identifies and names the colors of six blocks arranged in a specific sequence. The colors or positions of the blocks are then changed, and the child must reorganize them according to the new arrangement or create alternative sequences. Assemble and program robots with a different challenges.	LEGO^®^ DUPLO^®^ bricks WeDo 2.0^®^ Set WeDo 2.0^®^ Set/Spike essential Assembled blocks template Colored blocks of different size
(10) to (15) Integration of follow-up and executive functions integration	Cognitive habilitation and developmental monitoring	Six-piece memory kit assembly Arming and assembling the robot Exercise of progressive and regressive mathematical order Complex task to solve with the robot Classification of animals set, concrete, functional and abstract	LEGO^®^ DUPLO^®^ bricks Set SPIKE™ prime LEGO^®^ Education Animals Set LEGO^®^ Education More to Math Set

**Table 3:** BANFE-3 standardized total scores in ASD children (CTRL group).

Variable	Pre (Mean ± SD)	Post (Mean ± SD)	Post-Pre (Mean ± SD)
Subtotal OMC	79.0 ± 29.4	72.2 ± 27.3	−6.8 ± 24.1
Subtotal APC	79.1 ± 21.0	80.0 ± 27.0	0.9 ± 21.9
Subtotal DLC	67.7 ± 16.2	68.3 ± 17.5	0.6 ± 6.1
Total Executive Functions	66.8 ± 16.8	66.5 ± 17.0	−0.3 ± 9.2

ASD = Autism spectrum disorder. CTRL = control. OMC = orbitomedial cortex; APC = anterior prefrontal cortex; DLC = dorsolateral cortex.

**Table 4:** BANFE-3 standardized total scores in ASD children (LBN group).

Variable	Pre (Mean ± SD)	Post (Mean ± SD)	Post-Pre (Mean ± SD)
Subtotal OMC	76.5 ± 33.0	**86.6 ± 29.2**	12.5 ± 23.6
Subtotal APC	89.7 ± 32.9	85.0 ± 25.1	−3.4 ± 28.5
Subtotal DLC	65.7 ± 18.5	**83.3 ± 27.2**	17.9 ± 10.2
Total Executive Functions	66.4 ± 20.7	**81.9 ± 30.6**	18.5 ± 14.6

ASD = Autism spectrum disorder. LBN = LEGO^®^-based neurotherapy. OMC = orbitomedial cortex. APC = anterior prefrontal cortex; DLC = dorsolateral cortex. Increases in the BANFE-3 scores in the posttest are in bold.

## Data Availability

The data presented in this study are not publicly available because of ethical and privacy restrictions involving clinical pediatric populations but are available from the corresponding author upon reasonable request.

## Supplementary Materials

The following supporting information can be downloaded at https://www.mdpi.com/article/10.3390/brainsci16060633/s1: Table S1: ANCOVA and effect sizes for the Effect of LBN in children with ASD as evaluated by the BANFE-3 test; Table S2: Model diagnostic metrics and statistical tests for the ANCOVA assumptions; Table S3: Effect of LBN on the gain score of children with ASD, as evaluated by the BANFE-3 test.

## Abbreviations

The following abbreviations are used in this manuscript:

ASD	Autism Spectrum Disorder
LBT	LEGO^®^-Based Therapy
LBN	LEGO^®^-Based Neurotherapy
INP	Instituto Nacional de Pediatría
ICD-11	International Classification of Diseases, 11th Revision
ADOS-2	Autism Diagnostic Observation Schedule, Second Edition
ADI-R	Autism Diagnostic Interview-Revised
CARS-2	Childhood Autism Rating Scale, Second Edition
SRS-2	Social Responsiveness Scale, Second Edition
CTRL	Control
BANFE	Neuropsychological Battery of Executive Functions and Frontal Lobes
OMC	Orbitomedial Cortex
APC	Anterior Prefrontal Cortex
DLC	Dorso-Lateral Cortex
ANCOVA	Analysis of covariance
pwc	pairwise comparisons
