# Supplementary material for: Effects of LEGO®-Based Neurotherapy on Executive Functions in Children with Autism Spectrum Disorder: A Quasi-Experimental Study
Source: Brain Sci. 2026 Jun 12;16(6):633. doi: 10.3390/brainsci16060633 (PMC13296840; doi:10.3390/brainsci16060633)
Supplement: Supplementary file 1 [file brainsci-16-00633-s001.zip › brainsci-4323131-supplementary.pdf]

brain sciences

Supplementary Material

1. Effects of LBN on Children with ASD

Table S1: ANCOVA and effect sizes for the Effect of LBN in children with ASD as evaluated by the BANFE-3 test.

**Statistical Results**	**Subtotal OMC**	**Subtotal APC**	**Subtotal DLC**	**Total Executive Functions**
F values	F = 3.220<br>df1=1, df2=18,<br>p-value = 0.089	F = 0.019<br>df1=1, df2=18,<br>p-value = 0.891	F = 26.974<br>df1=1, df2=18,<br>p-value = 0.00006	F = 12.181<br>df1=1, df2=18,<br>p-value = 0.0026
Emmeans	Post-Pre = 1.790<br>p-value = 0.089	Post-Pre = 0.139<br>p-value = 0.891	Post-Pre = 5.190<br>p-value = 0.00006	Post-Pre = 3.49<br>p-value = 0.0026
Effect size: partial eta squared (η^2)<br>95% CI	$\eta^2 = 0.05$<br>(0.00, 1.00)<br>Magnitude= small	$\eta^2 = 0.0$<br>(0.00, 1.00)<br>Magnitude= small	$\eta^2 = 0.50$<br>(0.29, 1.00)<br>Magnitude= large	$\eta^2 = 0.34$<br>(0.08, 1.00)<br>Magnitude= large
Effect sizes: epsilon squared (ε^2)<br>95% CI	$\varepsilon^2 = 0.09$<br>(0.00, 1.00)<br>Magnitude= small	$\varepsilon^2 = 0.04$<br>(0.00, 1.00)<br>Magnitude= small	$\varepsilon^2 = 0.53$<br>(0.28, 1.00)<br>Magnitude= large	$\varepsilon^2 = 0.40$<br>(0.12, 1.00)<br>Magnitude= large

Table S2: Model diagnostic metrics and statistical tests for the ANCOVA assumptions.

**ANCOVA assumption Statistical Test**	**Subtotal OMC**	**Subtotal APC**	**Subtotal DLC**	**Total Executive Functions**
Reset linearity test	R = 0.355<br>df1=2, df2=16<br>p-value = 0.706	R = 0.447<br>df1=2, df2=16<br>p-value = 0.647	R = 0.842<br>df1=2, df2=16<br>p-value = 0.449	R = 0.169<br>df1=2, df2=16<br>p-value = 0.846
Rainbow linearity test	Rain = 2.25<br>df1=11, df2=7<br>p-value = 0.145	Rain = 1.526<br>df1=11, df2=7<br>p-value = 0.195	Rain = 0.529<br>df1=11, df2=7<br>p-value = 0.834	Rain = 0.566<br>df1=11, df2=7<br>p-value = 0.809
Homogeneity of regression slopes	F = 0.190<br>df1=1, df2=17<br>p-value = 0.669<br>Homogeneity	F = 0.778<br>df1=1, df2=17<br>p-value = 0.390<br>Homogeneity	F = 3.570<br>df1=1, df2=17<br>p-value = 0.0760<br>Homogeneity	F = 0.980<br>df1=1, df2=17<br>p-value = 0.336<br>Homogeneity
Shapiro-Wilk normality test	S = 0.938<br>p-value = 0.196	S = 0.939<br>p-value = 0.206	S = 0.913<br>p-value = 0.063	S = 1.000<br>p-value = 0.330
Levene Homoscedasticity test	F = 0.197<br>df1=1, df2=19<br>p-value = 0.662	F = 0.426<br>df1=1, df2=19<br>p-value = 0.522	F = 0.011<br>df1=1, df2=19<br>p-value = 0.918	F = 1.0<br>df1=1, df2=19<br>p-value = 0.330
Influence/outlier diagnostics	No outliers	No outliers	No outliers	No outliers

2. Analysis of the Gain Score.

Table S3: Effect of LBN on the gain score of children with ASD, as evaluated by the BANFE-3 test.

Gain-scores	Subtotal OMC	Subtotal APC	Subtotal DLC	Total Executive Functions
Median (1st, 3rd quartiles)	CTRL: 0.0<br>(-28.0, 6.0)<br>LBN: 8.0<br>(-0.5, 26.5)	CTRL: 0.0<br>(-13.0, 14.0)<br>LBN: 8.0<br>(-20.0, 1.5)	CTRL: 0.0<br>(-1.0, 1.0)<br>LBN: 17.0<br>(-12.0, 22.0)	CTRL: 0.0<br>(-4.0, 4.0)<br>LBN: 8.0<br>(-9.0, 26.0)
Wilcoxon rank sum test with continuity correction	W = 35.5<br>p-value = 0.1794	W = 68<br>p-value = 0.3784	W = 9<br>p-value = 0.0013	W = 15<br>p-value = 0.0051
Wilcoxon rank sum test	W = 35.5<br>p-value = 0.1682	W = 68<br>p-value = 0.3597	W = 9<br>p-value = 0.0012	W = 15<br>p-value = 0.0046
Effectsize	effsize = 0.301<br>n1=10, n2=11<br>magnitude = moderate	effsize = 0.200<br>n1=10, n2=11<br>magnitude = small	effsize = 0.709<br>n1=10, n2=11<br>magnitude = large	effsize = 0.619<br>n1=10, n2=11<br>magnitude = Large
R effectsize<br>95% CI	0.76<br>(-0.10, 1.63)	-0.17<br>(-1.1, 0.76)	1.43<br>(0.79, 2.07)	1.22<br>(0.48, 1.95)
Effectsize rc rank-biseral correlation<br>95% CI	-0.35<br>(-0.70, 0.13)	0.24<br>(-0.26, 0.63)	-0.84<br>(-0.94, -0.61)	-0.73<br>(-0.89, -0.39)

https://doi.org/10.3390/xxxxx
